# Pro-apoptotic and migration-suppressing potential of EGCG, and the involvement of AMPK in the p53-mediated modulation of VEGF and MMP-9 expression

**DOI:** 10.3892/ol.2013.1533

**Published:** 2013-08-19

**Authors:** SONG YI PARK, CHANG HEE JUNG, BOKYUNG SONG, OCK JIN PARK, YOUNG-MIN KIM

**Affiliations:** 1 Department of Biological Sciences and Biotechnology, Hannam University Daedeok Valley Campus, Yuseong-gu, Daejeon 305-811, Republic of Korea; 2 Department of Food and Nutrition, Hannam University Daedeok Valley Campus, Yuseong-gu, Daejeon 305-811, Republic of Korea

**Keywords:** p53, vascular endothelial growth factor, matrix metalloproteinase-9, migration-suppressing effects, epigallocatechin-3-gallate

## Abstract

The present study investigated the regulatory mechanisms by which epigallocatechin-3-gallate (EGCG) exerts vascular endothelial growth factor (VEGF)-, p53- and AMP-activated protein kinase (AMPK)-associated pro-apoptotic and migration-suppressing effects on colon cancer cells. EGCG decreased the expression levels of VEGF and matrix metalloproteinase (MMP)-9. EGCG treatment induced apoptosis in the presence of wild-type and mutant p53, indicating that a p53-independent pathway may contribute to EGCG-induced apoptosis in these cells. EGCG showed migration-suppressing effects, suggesting that this activity may also have p53-dependent and -independent components. The interaction between p53 and VEGF in the EGCG-treated cells was investigated using pifithrin-α. Notably, the suppression of p53 activity blocked the ability of EGCG to inhibit VEGF and MMP-9 in the cells expressing wild-type p53, but not mutant p53, indicating that the effects of EGCG on VEGF may be p53-dependent or -independent. Finally, although AMPK and VEGF did not appear to co-localize, the results indicated that AMPK controls VEGF in EGCG-treated cells regardless of the p53 status.

## Introduction

Colorectal cancer is a major cause of cancer-related mortality worldwide. The incidence of new cases has increased in recent years, particularly in affluent societies, and colon cancer is often highly metastatic and resistant to anticancer treatment strategies (1,2). One of the key genetic defects that confers resistance against colon cancer treatment is the mutation of p53 (3). This key apoptotic regulator and tumor suppressor mediates multiple responses to anticancer agents by modulating p21-associated cell cycle control, apoptosis and DNA repair (4). Mutations in p53 have been shown to abolish the efficiency of cancer treatment *in vitro* and *in vivo*, while reintroduction of wild-type p53 was demonstrated to sensitize p53-null cancer cells to therapeutic agents (5). *In vitro* and *in vivo* studies have suggested that a mutation in p53 synergistically interacts with hypoxia to elevate the expression of vascular endothelial growth factor (VEGF; a tumor-associated angiogenesis factor) in cancer cells. This is notable as VEGF is an important factor in cancer cell proliferation and metastasis, and p53 inhibits various cancer cell survival signals, including VEGF (6–9).

A number of phytochemicals have been reported to activate p53, thereby inducing apoptosis and suppressing the expression levels of VEGF and matrix metalloproteinase (MMP)-9, which are elevated in colon cancer (10–12). One such phytochemical, epigallocatechin-3-gallate (EGCG), the major polyphenolic compound of green tea, has been reported to have anti-proliferatory and apoptotic effects in a number of types of cancer cells (13). VEGF is known to support tumor growth and metastasis, and the status of VEGF has been shown to predict the prognosis of various human tumors (14). MMP-9 is one of the most important proteins involved in cancer cell metastasis and the status of MMP-9 has been shown to be overexpressed in various cancer cells (15). However, no previous study has examined the effects of EGCG on VEGF or MMP-9 and the involvement of p53 in this process.

The present study examined the effects of EGCG on VEGF and MMP-9 protein expression in two colon cancer cell lines: HCT-116 cells, which express wild-type p53, and HT-29 cells, which express mutant p53. The effects of EGCG on migration and apoptosis in the two cell lines and the possible involvement of AMP-activated protein kinase (AMPK) activation in the response to EGCG were also tested.

## Materials and methods

### Cells and reagents

The HCT116 and HT-29 human colon cancer cell lines were purchased from the American Type Culture Collection (Manassas, VA, USA) and were cultured in RPMI-1640 with 10% fetal bovine serum (Gibco, Rockville, MD, USA). EGCG, 3-(4,5-dimethylthiazol-2-yl)-2,5-diphenyltetrazolium bromide (MTT) and Hoechst 33342 were obtained from Sigma (St. Louis, MO, USA). Pifithrin-α and compound C were purchased from Calbiochem (San Diego, CA, USA). Monoclonal antibodies specific for p53 and AMPKα1 were purchased from Cell Signaling Technology (Beverly, MA, USA). VEGF and MMP-9 antibodies were purchased from Santa Cruz Biotechnology, Inc., (Santa Cruz, CA, USA) and the β-actin antibody was obtained from Sigma.

### Cell proliferation measurements and morphological examination

Cells seeded on 96-well microplates at 4×10^3^ cells/well were incubated with test compounds at 50 and 100 μm for 48 h. Following incubation with the test compound, the medium was removed and the cells were incubated with 100 μl MTT solution (2 mg/ml MTT in PBS) for 4 h. The samples were then solubilized in DMSO. The purple formazan dye, converted from MTT by viable cells, was quantified by absorbance at 560 nm. For the morphological examination, the cells were grown on 6-well plates, treated with flavonoids for 48 h and then examined under a light microscope (×400 magnification).

### Apoptosis detection

Apoptosis was measured using a FITC-Annexin V apoptosis detection kit (BD Pharmingen™, San Diego, CA, USA) or Hoechst 33342 chromatin staining dye. For Annexin V/PI staining following treatment with selenium, the cells were harvested by trypsinization, washed with ice-cold phosphate-buffered saline (PBS) and suspended in a binding buffer at a density of 1×10^6^ cells/ml. The cells were stained with Annexin V-FITC and propidium iodide (PI) and analyzed by flow cytometry (Becton-Dickinson Biosciences, Franklin Lakes, NJ, USA). To examine chromatin condensation, the cells were stained with 10 μM Hoechst 33342 for 30 min and fixed with 3.7% formaldehyde for 15 min. Changes in chromatin condensation were observed by fluorescence microscopy (Olympus Optical Co., Tokyo, Japan).

### In vitro wound healing assay

An *in vitro* wound healing assay was applied to determine cell mortality caused by EGCG. This assay was performed using a standard method (16) with certain modifications. Briefly, 1×10^5^ HCT116 and HT-29 cells were seeded on a 6-well plate in complete medium overnight to obtain a full confluent monolayer. Subsequent to 12 h of starvation, a 20-μl pipette tip was used to create a straight cell-free wound. Each well was washed twice with PBS to remove any debris. The cells were then cultured in serum-free medium in the absence or presence of 50–100 μM EGCG. The distances between the two edges of the scratch were analyzed quantitatively.

### Western blot analysis

Following starvation for 12 h in serum-free medium, the cells were seeded into 6-well plates and treated with test compounds. Total proteins were extracted using a RIPA lysis buffer [50 mM Tris-HCl (pH 8.0), 1% NP-40, 0.5% sodium deoxycholate, 150 mM NaCl and 1 mM PMSF] and subjected to western blot analysis with specific antibodies. The proteins were then visualized by enhanced chemiluminescence (Intron, Kyunggi, Korea) and detected using a LAS 4000 chemiluminescence detection system (Fuji, Tokyo, Japan).

### Immunofluorescence staining

The cells were seeded on a 12-well plate with cover glasses. Subsequent to treatment with 10–50 μm EGCG for 24 h, the cells were fixed in 3.7% formaldehyde for 20 min at room temperature (RT) and permeabilized in 0.2% Triton X-100 for 20 min at RT. Then cells were blocked with 1% bovine serum albumin for 1 h. The cells were then incubated overnight with the primary antibodies of AMPKα1 and VEGF. Subsequent to being washed, the cells were incubated with Alexa 546-conjugated anti-rabbit IgG and Alexa 488-conjugated anti-mouse IgG (both from Molecular Probes, Eugene, OR, USA) for 1 h at RT. The cell nuclei were then stained with 10 μM Hoechst 33342 for 10 min and observed using a confocal microscope (Carl Zeiss, Thornwood, NY, USA).

### Statistical analysis

Cell viability and migration rate data were statistically analyzed using an unpaired t-test (SPSS, Inc., Chicago, IL, USA). P<0.05 was considered to indicate a statistically significant difference.

## Results

### EGCG suppresses cell proliferation and induces apoptosis in HT-29 and HCT116 cells

**Figure 1:**
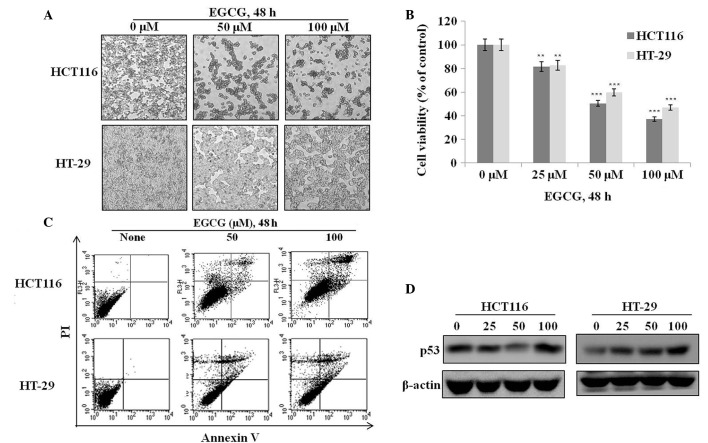
Effects of epigallocatechin-3-gallate (EGCG) on proliferation and apoptosis in HCT116 (wild-type p53) and HT-29 (mutant p53) colon cancer cells. (A) HCT116 and HT-29 cells were treated with or without various concentrations of EGCG for 48 h, and cell morphology was examined by light microscopy (×400). (B) Cell viability was determined by 3-(4,5-dimethylthiazol-2-yl)-2,5-diphenyltetrazolium bromide (MTT) assay, and is represented as the percentage of relative absorbance compared to controls. (C) Representative flow cytometric data from HCT116 and HT-29 cells that were treated with or without EGCG for 48 h and then stained with Annexin V and PI. (D) Cells were treated with various concentrations (25–100 μM) of EGCG for 24 h, and p53 protein expression was analyzed by western blotting. ^**^P<0.01 and ^***^P<0.001 vs. control (0 mM).

To investigate the effects of EGCG on cell proliferation and apoptosis, HT-29 and HCT116 cells were treated with various concentrations of EGCG for 48 h and their morphological, proliferative and apoptotic characteristics were observed. The majority of cells in the two EGCG-treated groups shrank and became globular in shape compared to the controls (Fig. 1A). An assessment of cell viability by MTT assay showed that EGCG dose-dependently suppressed the proliferation of the HT-29 and HCT116 cells (Fig. 1B). An examination of apoptosis by Annexin V staining showed that EGCG dose-dependently increased apoptotic cell death in the two cell types (Fig. 1C). These results suggest that the growth inhibitory properties of EGCG may arise from p53-dependent or -independent pathways. The ineffectiveness of EGCG in the regulation of mutated p53 (HT-29 cells) compared with the increment of p53 proteins with EGCG of HCT116 cells was shown (Fig. 1D). No previous study has clearly shown that EGCG induces apoptosis through both p53-dependent and p53-independent pathways. The present study reports that EGCG is capable of inducing apoptosis without using the traditional p53 pathway.

### EGCG possesses migration-suppressing potential

**Figure 2:**
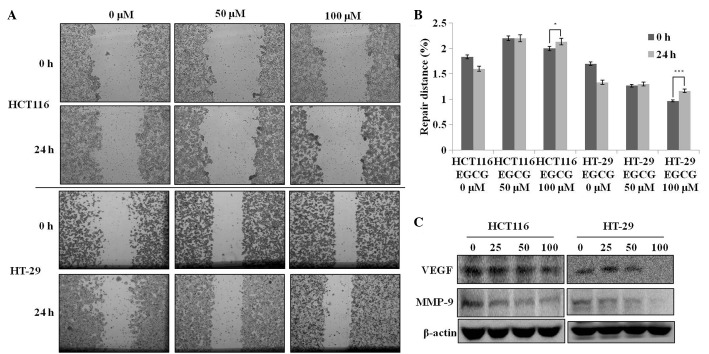
Effects of epigallocatechin-3-gallate (EGCG) on cell migration and the expression levels of vascular endothelial growth factor (VEGF) and matrix metalloproteinase (MMP)-9 in HCT116 and HT-29 colon cancer cells. (A) HCT116 and HT-29 cells were grown to confluence on a 6-well plate and then monolayers were wounded with a pipette tip and treated with EGCG or vehicle. Images of wound closure were captured under a phase-contrast microscope after 24 h. (B) The migration inhibition is presented as the distance between the edges of each scratch. (C) Cells were treated with different concentrations (25–100 μM) of EGCG for 24 h, and the protein expression levels of VEGF and MMP-9 were analyzed by western blotting. ^*^P<0.05 and ^***^P<0.001 for migration rates of 0 h and 24 h post-treatment with EGCG.

A wound-healing assay was used to evaluate the effect of EGCG on metastatic activity (i.e., migration). The treatment of the HCT116 and HT-29 cells with 50–100 μM EGCG for 24 h resulted in a significant reduction in the degree of wound healing (Fig. 2A), indicating that EGCG is able to inhibit metastatic activity in these two cell lines. Next, the present study tested whether the migration-suppressing activity of EGCG was associated with the attenuation of MMP-9 and VEGF protein levels. EGCG treatment dose-dependently decreased the protein expression levels of VEGF and MMP-9 (Fig. 2C). These results suggest that the migration-suppressing effect of EGCG is associated with the inhibition of VEGF and MMP-9 protein expression regardless of the p53 status in these cells.

### AMPK activation regulates VEGF and MMP-9 expression regardless of p53 status

**Figure 3:**
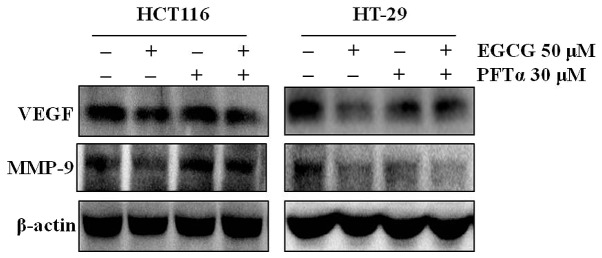
Vascular endothelial growth factor (VEGF) and matrix metalloproteinase (MMP)-9 are inhibited in epigallocatechin-3-gallate (EGCG)-treated HCT116 and HT-29 colon cancer cells. Cells were pretreated with 30 μM pifithrin-α for 30 min and then treated with 50 μM EGCG for 24 h. Proteins were subjected to western blot analysis using antibodies against VEGF and MMP-9.

To examine the involvement of p53 in the EGCG-induced inhibition of VEGF and MMP-9 expression, the HT-29 and HCT116 cells were treated with EGCG plus a specific inhibitor of p53 (pifithrin-α). Inhibition of p53 by pifithrin-α abolished the EGCG-induced inhibition of VEGF and MMP-9 in the HCT116 cells, but not in the HT-29 cells (Fig. 3).

## Discussion

**Figure 4:**
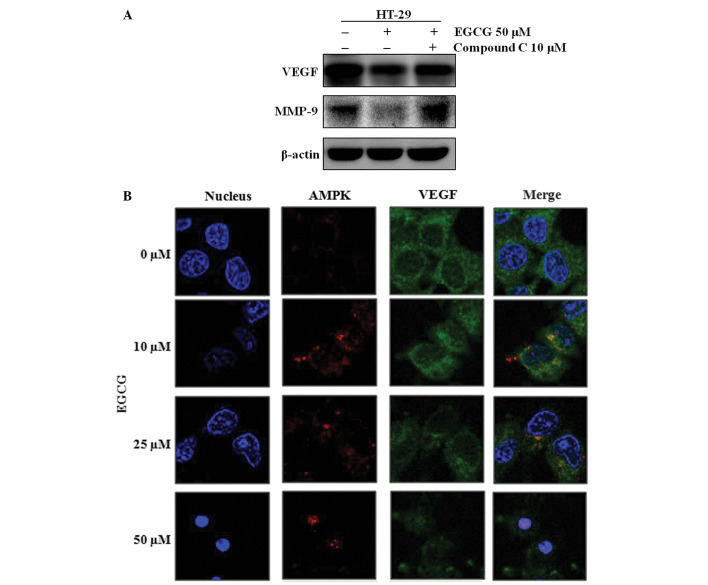
Epigallocatechin-3-gallate (EGCG) regulates vascular endothelial growth factor (VEGF) and matrix metalloproteinase (MMP)-9 expression through AMP-activated protein kinase (AMPK) in the absence of p53. (A) Cells were pretreated with 10 μM compound C for 30 min and then treated with 50 μM EGCG for 24 h. Proteins were subjected to western blot analysis using antibodies against VEGF and MMP-9. (B) Cells were treated with EGCG (10–50 μM) for 24 h, and then fixed, permeabilized and double stained with p-AMPKα1 and an anti-rabbit Alexa 546 secondary antibody (red) or with VEGF and an anti-mouse Alexa 488 secondary antibody (green). Cell nuclei were stained with Hoechst 33342 (blue) and the results were observed by confocal microscopy.

A previous study showed that the adenovirus-mediated gene transfer of wild-type p53 into p53-mutated cells is able to inhibit VEGF expression (17). Thus, the present results and those of the previous study indicate that p53 is involved in the ability of EGCG to inhibit VEGF and MMP-9 expression in p53-positive cells, but that EGCG is also able to regulate VEGF and MMP-9 via a p53-independent pathway in cells expressing mutant p53. Since other studies have suggested that EGCG activates AMPK (18) and inhibits VEGF (19), the EGCG-induced regulation of VEGF expression was tested in the presence and absence of the AMPK activity inhibitor, compound C. EGCG was no longer able to downregulate VEGF and MMP-9 in the compound C-treated HT-29 cells (Fig. 4A). Furthermore, EGCG activated AMPK and inhibited VEGF expression in a dose-dependent manner (Fig. 4B). Using an immunofluorescence analysis with AMPK- and VEGF-specific antibodies, it was observed that the regulatory effect of AMPK on VEGF did not result from their direct binding, since AMPK and VEGF did not co-localize (Fig. 4B). These results indicate that although AMPK does not directly bind VEGF, EGCG-activated AMPK controls VEGF.

In conclusion, the present study demonstrated that EGCG inhibits colon cancer cell migration and induces apoptosis in these cells regardless of the presence of functional p53. EGCG also strongly inhibits VEGF and MMP-9 expression in HT-29 (p53 mutant) and HCT116 (p53 wild-type) cells. This regulation appears to occur through p53 in cells expressing wild-type p53, but in p53-mutant cells this occurs via a p53-independent manner. Finally, although direct binding was not observed between AMPK and VEGF, the present results indicate that EGCG-induced AMPK activity regulates the expression levels of VEGF and MMP-9.
